# Traditional Chinese medicine Shenqi compound to improve lower extremity atherosclerosis of patients with type 2 diabetes by affecting blood glucose fluctuation: Study protocol for a randomized controlled multicenter trial

**DOI:** 10.1097/MD.0000000000019501

**Published:** 2020-03-13

**Authors:** Qianru Zhu, Jian Kang, Gang Xu, Jinyao Li, Hui Zhou, Ya Liu

**Affiliations:** Hospital of Chengdu University of Traditional Chinese Medicine, Sichuan, China.

**Keywords:** blood glucose fluctuation, individualized treatment based on syndrome differentiation, islet β-cell function, Shenqi compound, traditional Chinese medicine, type 2 diabetes with lower extremity atherosclerosis

## Abstract

**Background:**

Clinical and basic research supports that blood glucose fluctuation is an important predictor of diabetic vascular disease and an etiology of lower extremity atherosclerosis, which is an important pathological basis for lower extremity vascular diseases. Previous Chinese National Natural Science Foundation trials (No. 81503566) have reported that the traditional Chinese medicine Shenqi compound can reduce blood glucose fluctuation and low-grade inflammation, and protect blood vessels; however, there are no high-quality clinical evidences available to support the same. This multicenter randomized controlled trial aims to obtain more clinical evidence to confirm the efficacy and safety of Shenqi compound in type 2 diabetes with lower extremity atherosclerosis.

**Methods:**

A multicenter RCT will be implemented in this study for a 32-week study period (8 weeks for intervention and 24 weeks for follow-up). Participants will be recruited from the Teaching Hospital of Chengdu University of TCM, Mianyang Hospital of TCM, and Shuangliu Hospital of TCM. Sixty participants will be randomly divided into a treatment group (basic treatment combined with traditional Chinese medicine Shenqi Compound) or a control group (basic treatment combined with Chinese medicine placebo) with 30 participants in each group. Patients will be selected considering the following inclusion criteria: age between 35 and 65 years, and a positive diagnosis for type 2 diabetes with lower extremity atherosclerosis and TCM syndromes. Primary outcome indicator is an arterial color Doppler ultrasound. Secondary outcome indicators include: blood glucose fluctuation indicators (MBG, SDBG, LAGE), islet β-cell function evaluation indicators (Homa-IR, Homa-islet, SG, SCP), inflammation indicators (NLR, CRP, IL-6), blood lipids, and HbA1c. Safety index includes vital signs (T, P, R, BP), blood, urine, stool routine, liver and renal function, electrocardiogram, and adverse event records. The endpoint event is defined as the presence of gangrene in the lower limbs.

**Discussion:**

Explore the clinical effect of traditional Chinese medicine “Shenqi Compound” to reduce blood glucose fluctuation and use HOMA-IR, the area under the glucose curve, and the area under the C-peptide curve to evaluate the effect of protecting islet β cell function.

**Trial registration:**

: Chinese clinical trial registry (ChiCTR-1900027693). Registered on November 23, 2019. http://www.chictr.org.cn

## 1 Introduction

Recently, blood glucose fluctuations have gained significant attention with regard to type 2 diabetes research. Studies have reported that fasting and postprandial blood glucose fluctuation significantly increases in patients with type 2 diabetes,^[1]^ along with a remarkable increase in intra-day and intra-day blood glucose fluctuation, which are 6 and 2 mmol/L, respectively. Times and 2.5 times.^[2]^ Blood glucose fluctuation typically damages the vascular endothelial cells, increases the synthesis and secretion of adhesion molecules and abnormalities in their mediated adhesions, opens the hexosamine and polyol pathways, generates excess end-glycosylation products, and increases protein kinase C activity that consequently aggravates lower limb atherosclerosis.^[3]^ Blood glucose fluctuation is now considered to be an important predictor of chronic vascular complications associated with diabetes regardless of the presence of HbA1c,^[4]^ atherosclerotic heart, and brain, along with the lower extremity and other large blood vessels. The risk of disease is significantly higher for individuals with diabetes than in those without.^[5]^ Glucose fluctuation is one of the independent risk factors for macrovascular diseases, resulting in changes in oxidative stress and vascular endothelium damage, and it eventually leads to organ dysfunction and organic lesions.^[4]^ Methods to evaluate blood glucose fluctuation include standard deviation of blood glucose level, coefficient of variation of blood glucose, area under the blood glucose curve, maximum blood glucose fluctuation range, average blood glucose fluctuation range, and mean absolute difference in daily blood glucose.

## 2 Lower extremity atherosclerosis is an important pathological basis for vascular disease

Artherosclerosis (AS) is a vascular disease characterized by arterial intimal lipid deposition and calcification (plaque formation). There are generally no clinical symptoms in the early stages of the condition; however, the later stages demonstrate the presence of vascular occlusion and bilateral lower extremity ulcers. Type 2 diabetes with lower extremity atherosclerosis is the main cause of secondary foot ulcers and lower limb amputations in patients with diabetes, and is also a risk factor for coronary heart disease and cerebral infarction.^[6,7]^ Lower extremity arteries are prone to atherosclerosis due to their thickness and length, along with high blood pressure and endometrial injuries. Ultrasound is one reportedly of the first choices for the early diagnosis of lower extremity arterial disease in type 2 diabetes.^[8]^ It can observe a variety of indicators including the diameter of blood vessels, plaque distribution, and stenosis; furthermore, it judges the severity of lesions and provides a basis for treatment and prognosis.

## 3 Research basis of Shenqi compound and its unique hypoglycemic advantages

Shenqi compound has been used in clinical settings for 16 years. It was initially mass produced internally in the Teaching Hospital of Chengdu University of TCM in 2016. It has been used in 19 medical institutions in 19 districts (cities), counties, and high-tech zones within Sichuan province. Currently, it has been included in the “Diabetic Clinical Diagnosis and Treatment Program” of the National TCM Clinical Research (Diabetes) Base.

Previous research by the research group has confirmed that Shenqi compound can regulate glucose and lipid metabolism disorders, improve vascular function, and effectively prevent and treat diabetic vascular disease.^[9]^ In 2015, the National Natural Science Foundation of China (No. 81503566)^[10]^ found that it can regulate serum insulin level, inhibit mTOR activation and autophagy, and regulate the circadian clock Id2 and Usp2 genes of the pancreatic tissue to stabilize blood glucose. Comprehensive evaluation, Shenqi compound has multiple effects of reducing blood glucose fluctuation, reduces low-level inflammation, protects blood vessels, subsequently providing a research basis for this subject.

## 4 Methods

### 4.1 Objective

To compare the efficacy on reducing blood glucose fluctuation and improving lower extremity atherosclerosis of patients with type 2 diabetes of a combination of acarbose and insulin detemir, along with the effects of basic treatment of traditional Chinese medicine Shenqi compound.

### 4.2 Research type

Randomized controlled multicenter trial.

### 4.3 Screening of participants

The trial will be conducted at the Teaching Hospital of Chengdu University of TCM, Mianyang Hospital of TCM, and Shuangliu Hospital of TCM.

### 4.4 Groups

Participants will be divided into two groups: the treatment and control groups. Their basic treatment can maintain the original plan, which includes a hypoglycemic regimen comprising acarbose + insulin detemir, along with antihypertensive drugs, such as ACEI, ARB, diuretics, calcium antagonists, and β-blockers. Following which lipid-lowering drugs like atorvastatin will be administered.

Control group: Basic treatment + Chinese medicine placebo.

Treatment group: Basic treatment + Shenqi compound. One bag of the test drug three times a day, boiled with water before meals, stirred, covered, and sealed for 3 to 5 min, for a period of 4 weeks. Followed by observation for 8 weeks.

### 4.5 Interventions

#### 4.5.1 Arrangement for intervention

All researchers in this study will be trained beforehand. Participants will be instructed by trained researchers on how to use their medication and their involvement in the study.

#### 4.5.2 Lifestyle intervention

Subjects in both groups of the study will receive dietary, lifestyle, and exercise advice from researcher. This advice is according to the 2017 China Type 2 Diabetes Prevention Guide.

#### 4.5.3 Traditional Chinese medicine intervention

All participants in the treatment group will visit the researchers once every month, when the same Chinese doctor will examine them using four TCM diagnostic methods including inspection, listening and smelling, inquiry, and pulse measurement and palpation. Shenqi compound is composed of 8 Chinese herbs: ginseng, astragalus, raw radix rehmanniae, trichosanthin, dogwood, yam, salvia, and wine rhubarb. Ginseng and astragalus supplement the vitality together as the remedy. The four medicines are used in cooperation with each other to supplement the deficiency of yin and jin of the lungs, spleens, and kidney. Salvia miltiorrhiza and wine rhubarb are used as adjuvants to promote blood circulation and remove blood stasis. Shenqi compound regulates the body's energy metabolism and reduces blood glucose fluctuation, which is a good recipe for clinical treatment of type 2 diabetes with lower extremity atherosclerosis. At present, it has been included in the “Diagnosis and Treatment Program for Diabetes” of the Chinese Traditional Medicine Clinical Research (Diabetes) Base. The test drug is a Chinese medicine formula granule produced by Sichuan Green Pharmaceutical Technology Development Co., Ltd. Certification and quality control by director of the department of pharmacy department in the Teaching hospital of Chengdu University of TCM. Usage instructions suggest consuming the granules three times a day one lattice each time, by boiling with water before meals, stirring, covering, and sealing for 3 to 5 min. The main ingredients of placebo are beta-cyclodextrin and artificial food colors. Shenqi compound and placebo are both fried-free granules, are similar in packaging, appearance, and shape. Participants are not allowed to take other drugs during the 8-week study period. Each participant will receive 28 boxes of Chinese medicine formula granules. Participants will be asked to record the changes in their symptoms daily using a form reviewed by a researcher once every month.

#### 4.5.4 Randomization

The randomization sequence will be generated according to the PROCPLAN procedure statements of the SAS software package.

According to the 1:1 random arrangement of the treatment and control group, 30 cases in each group are categorized using random distribution cards, and the subjects are assigned to each group according to a predetermined random number.

#### 4.5.5 Allocation concealment

The random number table will be generated by a designated researcher using the SAS system, and the researcher will not participate in the selection or recruitment of participants.

#### 4.5.6 Blinding

Considering the unique characteristics of the TCM methods used in this trial, it would be difficult to blind the researchers administering the TCM treatments and the participants. However, the researchers responsible for the statistical analyses will be blind to the grouping information.

#### 4.5.7 Sample size

We aim to select a sample size estimation method when comparing the two sample means. The following calculation formula may be used when the sample number of the test group and the control group is expected to be equal:
 
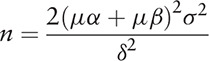


Here, n represents the estimated sample size of each test group, μα and μβ are the μ values corresponding to α and β, where μα represents the μ value of the probability of a type I error, μβ represents the μ value of a type II error, and δ is consistent with the required degree of discrimination, and σ is the standard deviation of the population. In the formula, μα and μβ can be calculated from the degree of freedom table of the *t*-boundary value υ = ∞−. If the test is required to have a probability of making a type I error >5% and a probability of making a type II error >10%, that is 0.05, β = 0.1, t value table indicated that *t*0.05 = 1.64, *t*0.1 = 1.28, refers to the relevant Chinese medicine treatment of type 2 diabetes blood sugar fluctuations, diabetes combined with lower extremity atherosclerosis.^[11,12]^ The standard deviation of indicator of blood glucose fluctuation–blood glucose (SDBG) in the literature will be set as the clinical efficacy observation, with SDBG <2.0 mmol/L being normal, then σ = 2.41, δ = 2.0. Here, the value of n brought into the formula is about 24.76, so the minimum sample size for this test is 50 cases. The estimated lost follow-up rate is 20%, and the final number of cases included is 60.

### 4.6 Patient identification and enrollment

#### 4.6.1 Source of participants

The participants will be recruited through the outpatient and inpatient departments of the Teaching Hospital of Chengdu University of TCM, Mianyang Hospital of TCM, and Shuangliu Hospital of TCM. We will also include 40 cases of Teaching Hospital of Chengdu University of TCM, 10 cases of Mianyang Hospital of TCM and 10 cases of Shuangliu Hospital of TCM.

#### 4.6.2 Diagnostic criteria

(1) Western medicine diagnosis and classification criteria for diabetes: the criteria recommended in the 1999 WHO expert consultation report.^[13]^

Diagnostic criteria: Patients conforming to one of the following conditions can be diagnosed

- Symptoms of diabetes + plasma glucose level at any time ≥11.1 mmol/L (200 mg/dL)
- Fasting plasma glucose (FPG) level ≥7.0 mmol/L (126 mg/dL)
- In the glucose tolerance test, the plasma glucose level at 2 h is ≥11.1 mmol/L (200 mg/dL)

Classification criteria:

- Type 1 diabetes
- Type 2 diabetes
- Other typing (omitted)

Patients who meet one of the conditions can be diagnosed with diabetes. This study targeted patients with type 2 diabetes and excluded those with type 1, gestational, and other special variations of diabetes.

(2) Lower extremity atherosclerosis diagnostic criteria: “Specifications for the screening and management of lower extremity arterial disease in patients with type 2 diabetes” issued by the Chinese Medical Association Diabetes Branch in 2013. Imaging basis: according to the color Doppler ultrasound images, the measured lower extremity blood vessels include the common iliac artery, external iliac artery, superficial femoral artery, iliac artery, anterior tibial artery, peroneal artery, posterior tibial artery, and dorsal foot artery. Lower extremity atherosclerosis is considered if the medial thickening of the wall of any blood vessels ≥1 mm, it is not smooth, atherosclerotic plaques are present, or the local lumen of the artery is narrowed or even completely occluded. Detection of lower limb IMT: <1.0 mm is considered normal, 1.0 to 1.2 mm is considered mildly thickened, >1.2 mm is considered to be an atheromatous plaque formation, while the presence of a normal and mild thickening suggests the absence of an atheromatous plaque, subsequently leading to the diagnosis of lower extremity atherosclerosis.^[14]^

(3) TCM syndrome diagnostic criteria for diabetic Qi-yin deficiency and blood stasis syndrome: according to the “ Guidelines for clinical research of new Chinese medicines.”^[15]^

- Main symptoms: dry mouth, dry throat, fatigue, numbness, or tingling in the limbs
- Secondary symptoms: shortness of breath and words, hot hands and feet, constipation, yellow urine
- Tongue image: red and dry, dark color with blood stasis, sublingual veins are dark
- Pulse: weak, thin and fast, or blocked or string
  1. Diagnosis can be made considering the tongue and pulse.
  2. It can be diagnosed with the presence of dry mouth and throat, fatigue in combination with dark ecchymosis of the tongue or bruising purple veins or astringent pulses.

#### 4.6.3 Inclusion criteria

Participants are considered eligible for the study if they meet the following criteria:

1. Voluntarily participation in the trial with signature on the informed consent form;
2. Aged 35 to 65 years;
3. Diagnosis of Western medicine type 2 diabetes and TCM syndrome, and diabetic macrovascular complications

#### 4.6.4 Exclusion criteria

1. Patients with heart and kidney dysfunction, acute coronary syndrome, cerebrovascular accident, hematopoietic disorders, and mental illness;
2. Patients with acute metabolic disorders such as diabetic ketoacidosis within the past 1 month;
3. Pregnant or lactating women;
4. Severe infection in the past 1 month;
5. Patients with allergic constitution;
6. Uncooperative in run-in period;
7. Having a history of alcoholism or drug abuse

#### 4.6.5 Rejection criteria

1. Those who do not to meet either the inclusion or exclusion criteria after enrollment;
2. Those who have not used the medicines according to the regulations, cannot judge the curative effect or provide incomplete information;
3. Those who have consumed drugs that are forbidden and may influence the judgment of effection;
4. Those who have acute infection during the test

#### 4.6.6 Exit criteria

1. Poor effection, along with the subject not following through with the complete treatment course;
2. Presence of adverse reactions that are difficult to tolerate, resulting in the subject leaving the study;
3. Occurrence of serious adverse events (AEs);
4. Complications associated with other diseases during the trial deeming the participant unsuitable to continue the test;
5. Subjects withdrawing from the trial without an explanation

#### 4.6.7 Suspension criteria

1. Occurrence of serious safety problems during the test;
2. Suspension ordered by the administrative department.

Researchers should strive to contact the participants to ascertain the reasons for dropping out from the study, and to acquire other relevant information such as the last time that they had taken the decoction, and to convince them to finish the examinations as far as they possibly can. If the participants experience any AEs during the study, researchers should provide appropriate treatment for them. The expected dropout rate should be <15%. The dropout cases will be included in the statistical analysis.

### 4.7 Follow-up

#### 4.7.1 Participant timeline

**Figure 1:**
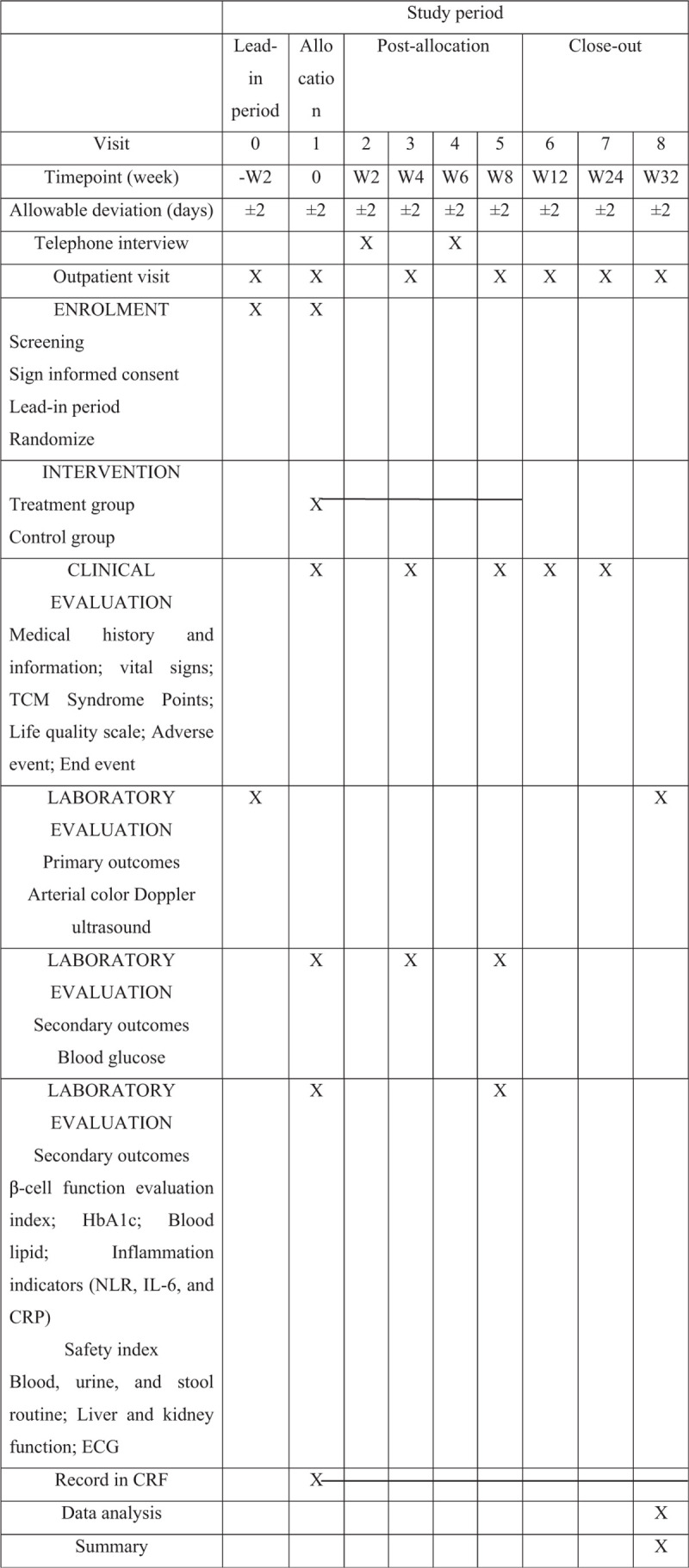
The schedule of visits and measurements.

Screening, intervention, and assessment visits will be performed by a researcher. The schedule of visits and measurements is given in Figure 1 in compliance with Standard Protocol Items: Recommendations for Interventional Trials (SPIRIT) figure.

Screening period: 2 weeks prior to the intervention. Intervention period: 8 weeks. Follow-up period: 24 weeks after the intervention.

### 4.8 Outcome measures

#### 4.8.1 Primary outcomes

1. Arterial color Doppler ultrasound. (Assess arterial intimal thickness, degree of sclerosis, plaque, stenosis)^[16,17]^

Arterial color Doppler ultrasound will be tested at Visits 0 and 8.

#### 4.8.2 Secondary outcomes

1. Measurement of blood glucose levels before three meals, 2 h after three meals, and before sleeping; calculation of average blood glucose levels (MBG), standard deviation of mean blood glucose (SDBG), and maximum blood glucose fluctuation (LAGE).
2. β-cell function evaluation index: insulin resistance index (HOMA-IR), area under the glucose curve (SG), and area under the C-peptide curve (SCP). Calculating HOMA-IR, SG, and SCP by:
  Homa-IR (CP) = 1.5 + G 0 min × CP 0 min/2800; Homa-islet (CP) = 0.27 × CP 0 min/(G 0 min − 3.5);
  SG = (G 0 min + G 180 min)/2 + G 30 min + G 60 min + G 120 min;
  SCP = (CP 0 min + CP 180 min)/2 + CP 30 min + CP 60 min + CP 120 min;
  (G 0 min to G 180 min, CP 0 min to CP 180 min, respectively means blood glucose and C-peptide values at each time point after Steamed bun test).^[18,19]^
  Blood glucose will be tested at visits 1, 3, and 5. β-cell function evaluation index will be tested at visits 1 and 5.
3. Blood lipid: total cholesterol (TC), triglyceride (TG), high density lipoprotein cholesterol (HDL-C), and low density lipoprotein cholesterol (LDL-C);
4. Inflammation indicators (NLR, IL-6, and CRP)
5. HbA1c

Blood glucose will be tested at visits 1, 3, and 5. β-cell function evaluation index, HbA1c, inflammation indicators and blood lipid will be tested at visits 1 and 5.

#### 4.8.3 Life quality index

Using the assessment scale for the patient's quality of life (SF-36 scale Chinese version) is used.

All life quality assessment scales will be tested at visits 1, 3, 5, 6, and 7.

#### 4.8.4 Safety index

Vital signs (T, P, R, BP), blood, urine, and stool routine, liver function (ALT, AST, TBIL, AKP, GGT), renal function (BUN and CR), ECG.

All safety indexes will be tested at visits 1 and 5.

#### 4.8.5 Endpoint event incidence

Select an indicator including the endpoint event incidence rate and time required to reach the endpoint event. The endpoint event is defined as the occurrence of gangrene of lower limbs.

**Figure 2:**
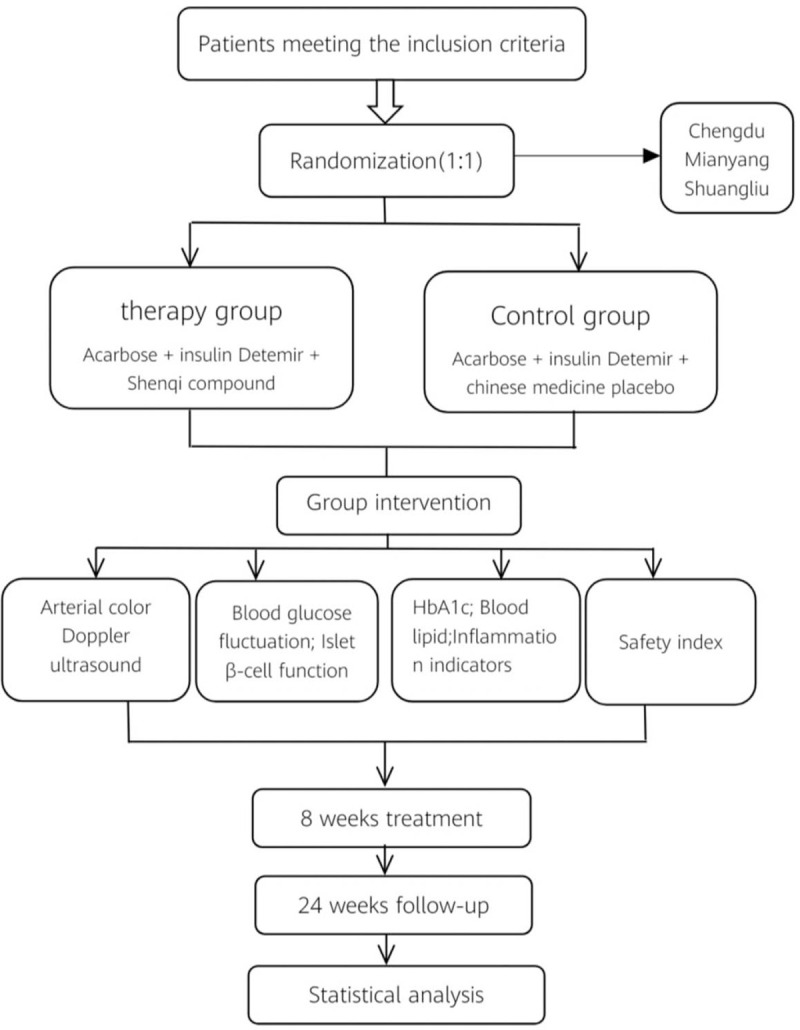
The technical route of the whole multicenter RCT.

Figure 2 shows the technical route of the whole multicenter RCT.

### 4.9 Data collection

#### 4.9.1 Clinical examination

Before the start of the trial, a history of diabetes, current medication, other medical history, and treatment history will be recorded. The anthropometric data will be collected at five time points (baseline, w4, w8, w12, w24), will be collected using standardized inspection procedures including: gender, age, height (shoes removed), weight (after morning urine, fasting status), body mass index (BMI), waist circumference (perimeter of the horizontal plane at the midpoint of the axillary midline from the rib arch to the midpoint of the crotch margin), hip circumference (largest possible hip circumference), waist-to-hip ratio (WHR). Measure vital signs including blood pressure, heart rate, breathing, and temperature.

#### 4.9.2 Blood samples

Blood samples will be collected after fasting overnight, including blood glucose levels (baseline, w4, w8), postprandial C peptide and glucose levels at 0.5, 1, 2, and 3 h (baseline, w8), along with HbA1c and blood lipid (baseline, w8). The kit is provided by Depp. Blood samples will be sent to the laboratory of Teaching Hospital of Chengdu University TCM for analysis.

#### 4.9.3 Urine samples

Urine samples will be collected (baseline, w8) using a urine sample collection container and will be sent to the laboratory of Teaching Hospital of Chengdu University TCM for analysis.

#### 4.9.4 Stool samples

Stool samples will be collected (baseline, w8) using a stool sample collection container and will be sent to the laboratory of Teaching Hospital of Chengdu University TCM for analysis.

#### 4.9.5 Adverse events

Adverse events in this study do not include diabetes-related complications including acidosis, water-electrolyte imbalance, and fever recorded in the medical records. The severity of all AEs should be judged, and the determination result should be recorded in the CRF, and the process associated with the AEs should be recorded. The severity of AEs is determined as follows:

Mild AEs: Patients have conscious symptoms and mild reactions, which are tolerable and do not affect normal activities. Symptoms are transient and resolve independently between cycles of medications or during treatment;
Moderate AEs: Affects the patient's normal daily activities, the symptoms last for a long time, and may interfere with treatment, and may require immediate intervention by either changing the dose or reducing the number of medications;
Severe AEs: Patients with impaired body functions, loss of normal work and living ability, symptoms lasting longer, indicating the need to discontinue the treatment

#### 4.9.6 Documentation of adverse events

The AE Report Form must be completed according to the circumstances. Some additional information, including the event time, severity, duration, measure adopted, and the outcome should be noted as well.

#### 4.9.7 Relationship between adverse event and study treatments

The researchers will assess the relationship between the AE and treatments being studied using the following criteria: appearance of the suspected adverse reaction after administering the treatment; whether the suspected adverse reaction belongs to the known adverse reactions of the investigated drug; whether the suspected adverse reaction disappears after discontinuation of treatment; whether the same reaction reoccurs again after resuming the investigated drug; and whether the suspected adverse reaction cannot be explained by the participant's diseases or consumption of the combination of drugs.

#### 4.9.8 Statistical analysis method

We will use the SPSS20.0 software for statistical analysis of the experimental data. Measurement data will be tested by *t* test, expressing as mean ± standard deviation. Paired design *t* test may also be used for intra-group comparisons. The chi-square test will be used for the enumeration data. Rank-sum test will be used for rank data. The comparison of survival rate and survival time between the two groups will be analyzed by Kaplan–Meier curve.

The analysis data set will be selected from the following:

1. Full analysis set (FAS): Including all subjects who received at least one treatment after randomization and having more than one follow-up record. If the case fails to complete the treatment course, the final observation should be implemented instead. The number of subjects who will undergo evaluation for the efficacy at the end point should be the similar to that at the beginning of the trial.
2. Per protocol (PP): In accordance with the treatment protocol, the main variables can be determined, and there is no major violation of the experimental protocol.
3. Statistical analysis of the main variables: Analyze the data of the full analysis set and the matching plan set.

#### 4.9.9 Document conservation and summary

Documents, such as the ICFs, signatures of participants and others need to be retained by researchers after the study according to Good Clinical Practice (GCP). Researchers should retain the clinical trial material for minimum 5 years.

### 4.10 Trial management

#### 4.10.1 Data management

We will use RRMS (http://www.medresman.org/login.aspx) to manage the trial data. RRMS can automatically generate an electronic Case Report Form (eCRF) for each case. Access to the eCRF password will be protected by administrator of the study on RRMS. All data will be entered into RRMS and can be checked publicly. The data will be independently and consistently entered by two personnel (double data entry). Following which, any changes in the same can be tracked by RRMS to ensure data accuracy. All data will be checked by researchers.

#### 4.10.2 Traditional Chinese medicine management

Participants will receive formula granules of Shenqi compound produced by Sichuan Green Pharmaceutical Technology Development (fixed prescription granules). Usage: one bag at a time, three times a day. Boil with water before meals, stir, cover, and seal for 3 to 5 min before consumption.

#### 4.10.3 Protocol management

Change in protocol: all changes in protocol will be documented, and any modifications of the protocol, including informed consent, must require the approval of the Ethics Committee.

Case Report Form (CRF) tracking: all signed ICFs must be submitted before the participants are included. Any questions or comments about CRFs must be submitted directly to the researchers.

#### 4.10.4 Request for researchers

Researchers in this trial must possess the qualifications and ability needed to conduct the research and will not be continually changed.

#### 4.10.5 Security

Researchers should carefully maintain participants’ personal information. Participant data in the CRF can only be accessed by related researchers and administrators.

## 5 Discussion

Although there has been a significant degree of progress with regard to the treatment of diabetic lower extremity vascular disease, the phenomenon of blood glucose fluctuation remains the highlight and the most challenging aspect of the treatment procedure. Reducing blood glucose fluctuations is a key issue in intervening diabetic vascular complications, especially lower extremity vascular disease, and reducing the morbidity and mortality of diabetic patients. Animal experiments have proven that Shenqi Compound has a clear therapeutic effect on diabetic macroangiopathy,^[20]^ which has gradually drawn our attention to clinical trials. In addition, the previous National Natural Science Foundation project study (No. 81503566) reported that Shenqi compound protected the islet β cells by inhibiting mTOR-activated autophagy, and regulating the expression of circadian rhythm genes in the pancreas to stabilize blood glucose.^[10]^

Compared to other studies, this study focuses on influencing blood glucose fluctuations and improving lower extremity atherosclerosis. Here, we want to further study the efficacy of Shenqi Compound in influencing the blood glucose fluctuation in type 2 diabetes with lower extremity atherosclerosis. Since blood glucose fluctuations are prone to damage the vascular endothelium and are a risk factor for lower extremity atherosclerosis,^[21]^ the effect of Shenqi Compound on arterial color Doppler ultrasound is evaluated as the main outcome indicator. Because blood glucose fluctuations are important predictors of vascular complications of diabetes,^4^ the evaluation of blood glucose fluctuations and islet β-cell function indicators will be calculated by formulae as secondary outcome indicators. Since HbA1c variability represents long-term blood glucose fluctuations,^[3]^ the effect of Shenqi compound on glycated hemoglobin is also evaluated. Furthermore, blood glucose fluctuation and lower extremity atherosclerosis are closely related to disorders of lipid metabolism,^[22]^ it is also imperative to evaluate the effects of Shenqi compound on blood lipids. Inflammatory cells play an important role in the occurrence and development of lower extremity atherosclerosis, due to which NLR is closely related to the formation of AS, subsequently highlighting the need to the effect of Shenqi compound on inflammation indicators, specifically NLR. Evaluating the severity of lower extremity atherosclerosis by detecting NLR clinically would be essential in providing a powerful reference for the refinement and management of patients.^[7]^ Bias will be minimized by adopting a rigorous methodology. Despite the fact that this study is an open label trial, some measures will be considered to strengthen quality control, considering the nature of RCT multicenter trial and CER design. To avoid the bias from the researchers, an independent investigator will be assigned in each research center as the contact person to preserve and record the randomization information. Therefore, clinical researchers will have no influence on enrollment or randomization. This study strives to effectively improve the blood glucose fluctuation and delay type 2 diabetes with lower extremity atherosclerosis, which has significant social benefits.

This study also has some limitations. First, the sample size is not large enough. Second, the 8-week treatment period and the 24-week follow-up period may slightly short. Further research can be carried out in the future for in-depth evaluation.

Summarily, this study provides the experimental basis for the clinical treatment of blood glucose fluctuation. The application of Chinese medicine Shenqi compound in type 2 diabetes with lower extremity atherosclerosis can obtain evidence-based medicine.

## Acknowledgments

We would like to thank Editage (www.editage.cn) for English language editing.

## Author contributions

**Data curation:** Jinyao Li.

**Formal analysis:** Hui Zhou.

**Investigation:** Gang Xu, Jinyao Li.

**Methodology:** Gang Xu, Hui Zhou.

**Project administration:** Ya Liu.

**Resources:** Ya Liu.

**Writing – original draft:** Qianru Zhu, Jian Kang.

**Writing – review & editing:** Qianru Zhu.
